# Yiqi Huoxue Recipe Delayed Intervertebral Disc Degeneration by Activating Autophagy

**DOI:** 10.3389/fphar.2021.705747

**Published:** 2021-08-18

**Authors:** Feng Dai, Pengfei Yu, Zhenhan Yu, Hong Jiang, Zhijia Ma, Jintao Liu

**Affiliations:** Department of Orthopedics, Suzhou TCM Hospital Affiliated to Nanjing University of Traditional Chinese Medicine, Suzhou, China

**Keywords:** yiqi huoxue recipe, autophagy, intervertebral disc degeneration, Beclin1-VPS34 complex, inflammatory cytokines

## Abstract

Autophagy has been proved to occur in rats with intervertebral disc degeneration (IVDD). Yiqi Huoxue recipe (YQHXR), an effective therapy of traditional Chinese medicine, was widely used for ruptured lumbar disc herniation under clinical observation. More importantly, YQHXR positively regulated the expression of autophagy-related proteins. However, little is known about the significance of YQHXR in the pathologic process of IVDD. Therefore, this study explored the protective effect of YQHXR based on IVDD rat model through magnetic resonance imaging and histopathologic analysis. Then we evaluated the formation of autophagosomes in the degenerated intervertebral disc by transmission electron microscope. Real-time PCR was used to detect the changes of autophagy-related genes. Western blot and immunoprecipitation were used to assess the protein expression of the autophagy-related pathway. We found that YQHXR-induced autophagy attenuated the release of inflammatory factors. In addition, YQHXR promoted the formation of Beclin1-VPS34 complex to activate autophagy through not only activation of the upstream protein AMPK and upregulation of the deubiquitinase USP13, thus in turn alleviating the development of IVDD. We proposed the potential molecular mechanism of YQHXR on autophagy for the first time, so as to provide a theoretical and experimental basis for the clinical application of YQHXR in the treatment of IVDD-related diseases.

## Introduction

Intervertebral disc degeneration (IVDD) is known as a common disease closely related to accelerated or advanced signs of aging, responsible for several spine-related disorders, such as disc herniation, chronic low back pain, and spinal canal stenosis (Adams and Roughley, 2006; Tang et al., 2018). The intervertebral disc consists of three parts: the nucleus pulposus (NP), the outer annulus fibrosus (AF), and the upper and lower cartilage endplates (Lin et al., 2020). The characteristics of IVDD are complex, with pathological changes in NP and inner ring, forming cracks radiating from the central area of the intervertebral disc to the periphery (Fraser et al., 1993). The clinical symptoms of IVDD are mainly based on the degenerative changes of the intervertebral disc, leading to long-term lumbago and leg pain, neurological deficits, and loss of labor ability, which seriously affect the life quality of patients (Xiong et al., 2020). Caused by accumulation of injury on the basis of degenerative diseases, IVDD will further repeatedly attack the degeneration of the intervertebral disc.

Excessive destruction of the extracellular matrix has been confirmed to be involved in the process of IVDD (Ding et al., 2013). Autophagy is a self-protection mechanism by which cells maintain stability by degrading their own aging substances, such as macromolecular aggregates, long-lived proteins, and damaged intracellular organelles (Rajawat and Bossis, 2008). Recently, autophagy has been proved to occur within the degenerative intervertebral discs of rats. Studies have shown that nucleus pulposus cells enhanced their viability to clear damaged tissues or organs through autophagy under various adverse stimuli, indicating that autophagy plays a critical role in protecting the survival of nucleus pulposus cells and delaying the occurrence of IVDD (Ma et al., 2013; Chen et al., 2014).

It is challenging to explore an effective therapy for IVDD, because of its complicated interaction with vascular, immunologic, and fibrotic components. After years of clinical practice, combined with traditional theory and modern medicine, we put forward a therapeutic principle of “Yiqi Huoxue”. Based on the theory of Traditional Chinese Medicine (TCM), “Yiqi Huoxue Recipe’’ (YQHXR) exerts its role by invigorating Qi, activating blood circulation, removing blood stasis, and dredging collaterals (Wang and Jiang, 2017 (in Chinese)). Previous studies have shown that YQHXR has the efficacy of anti-fibrosis, promotes ulcer healing, attenuates the formation of cerebral vascular microemboli, and improves the outcomes of ischemic stroke (Wu et al., 2014; Jiang et al., 2020; Ren et al., 2020). In addition, it was found in the kidney injury model, YQHXR activated autophagy and regulated the expression of autophagy-related proteins (LC3II and Beclin1), which offered renoprotection for rats with adenine-induced kidney disease (Xia et al., 2019). At present, this prescription has been widely used in clinics. The clinical observation of 102 cases confirmed that YQHXR is beneficial to the resorption of ruptured lumbar disc herniation (Yu et al., 2015).

All data clarified a potential role of YQHXR in the prevention and treatment of IVDD, but little is known about the significance of YQHXR in the pathologic process of IVDD. Therefore, this study explored the protective effect of YQHXR on the degenerative intervertebral disc through promoting autophagy, thus providing the scientific basis for the clinical application of YQHXR in the treatment of IVDD.

## Materials and Methods

### Composition and Preparation of Yiqi Huoxue Recipe

YQHXR is composed of Pheretima aspergillum (Di Long) and six herbs. *Astragalus mongholicus* Bunge (Huang Qi), *Stephania tetrandra* S.Moore (Fang Ji), *Ligusticum striatum* DC. (Chuan Xiong), *Angelica sinensis* (Oliv.) Diels (Dang Gui), and *Clematis chinensis* Osbeck (Wei Lin Xian) were provided by Suzhou chunhuitang Pharmaceutical Co., Ltd. (Jiangsu, China). *Brassica rapa L*. (BaiJie Zi) was provided by Suzhou Boyuan Pharmaceutical Co., Ltd. (Jiangsu, China). *Carica papaya L.* (Mu Gua) and Pberetima were provided by Suzhou chunhuitang Pharmaceutical Co., Ltd. (Jiangsu, China). The formula compositions are listed in Table 1.

**TABLE 1:** Main materials in Yiqi Buxue Recipe.

Chinese name	Latin name of medicinal material	Material	Batch number	Dose ratio (g)^a^
Huang Qi	*Astragalus mongholicus* Bunge	Root	190,819	20
Dang Gui	*Angelica sinensis* (Oliv.) Diels	Root	190,729	10
Chuan Xiong	*Ligusticum striatum* DC.	Rhizome	190,713	15
Di Long	*Pheretima aspergillum*	Body	191,224	15
Fang Ji	*Stephania tetrandr*a S.Moore	Root	190,924	10
Mu Gua	*Carica papaya* L	Root	200,104	10
Bai Jie Zi	*Brassica* rapa L	Seed	190,917	6
Wei Lin Xian	*Clematis chinensis* Osbeck	Roots and rhizomes	191,118	10

a Dry weight of the medicinal material.

All medicinal materials in YQHXR were weighed accurately according to the proportion in Table 1 and added into 5 times cold water to soak them for 30 min 200 ml of filtrate solution was collected through the filter screen. Then, the remaining solution was decocted with 300 ml of water for 30 min and 200 ml of filtrate was also collected through the filter screen. After filtration, two times of water extract were concentrated into 1 g crude drug/ml (final concentration) and stored in a 4°C refrigerator, which was prepared for experimental application. According to the conversion table of equivalent dose ratio between human and animal body surface area, the dosage of intragastric administration to rats was about 10 g/kg in the animal experiment.

### Quantitative Analysis by Ultra Performance Liquid Chromatography (UPLC)

**FIGURE 1:**
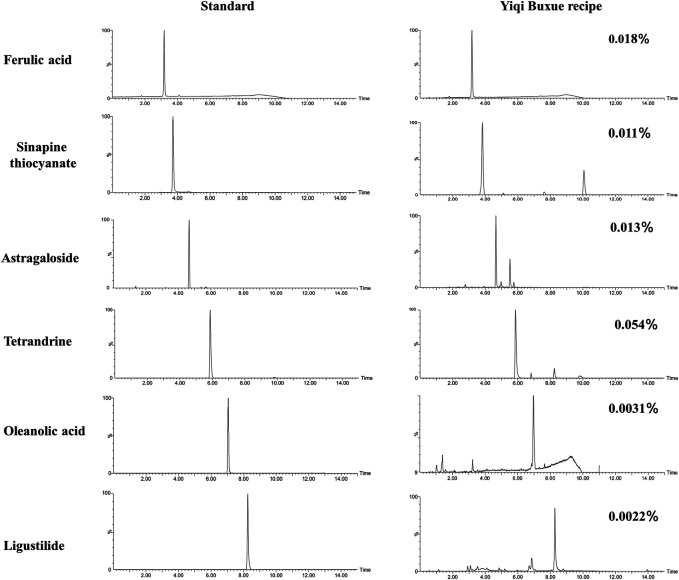
Representative chromatograms and relative content of Ferulic acid (RT, 3.20 min, content of 0.018%), Sinapine thiocyanate (RT, 3.76 min, content of 0.011%), Astragaloside (RT, 4.65 min, content of 0.013%, Tetrandrine (RT, 4.65 min, content of 0.054%), Oleanolic acid (RT, 7.12 min, wavelength at 274 nm, content of 0.0031%) and Ligustilide (RT, 8.24 min, content of 0.0022%) in YQBXR analyzed by UPLC.

In order to quantitative determination of the main compounds of YQHXR, chromatographic analysis was carried out in a Waters Acquity UPLC system (Waters, United States). Chromatographic separation was performed on an ACQUITY UPLC C18 column (100 × 2.1 mm, 1.7 μm) at 35°C. The mobile phase consisted of two solvents as follows: A solvent with water containing 0.1% formic acid and B solvent with acetonitrile containing 0.1% formic acid). The procedure of linear gradient elution: 8–30% B solvent at 0–6 min, 30–95% B solvent at 6–11 min, 95–99% B solvent at 11–14 min. The content of active ingredients was determined according to the standard curve established by the corresponding standard (Ferulic acid, Sinapine thiocyanate, Astragaloside, Tetrandrine, Oleanolic acid, and Ligustilide), respectively. The representative chromatography and relative content of active ingredients was depicted in Figure 1.

### Animal

Male Sprague-Dawley (*n* = 140) rats, weighing 190–200 g, were randomly assigned to different 28 cages. The temperature was maintained under an appropriate experimental condition (temperature of 24 ± 1°C, humidity of 50%, and the circadian rhythm for 12 h). All procedures in the animal experiments were approved by the Institutional Research Animal Ethical Committee of Suzhou Hospital of Traditional Chinese Medicine.

### Establishment of a Rat Model of Intervertebral Disc Degeneration

After rats were anesthetized with 5% isoflurane, surgical procedures were performed under aseptic conditions (Xu et al., 2017; Zamora et al., 2017). The specific location of the intervertebral disc between the fourth and fifth caudal vertebrae (C4-C5) was determined by palpation and radiology. Then, surgery was performed with a needle which puncture vertically at a depth of 5 mm through the tail skin and rotated 360° in the intervertebral disc for 30 s.

### Experimental Design

Animal experiment 1): Rats were randomly assigned to four groups (*n* = 10 per group): Sham group, IVDD group, IVDD + YQHXR-L group, IVDD + YQHXR-H group. After induction of the IVDD model described above, rats in IVDD + YQHXR-L and IVDD + YQHXR-H groups were treated with intragastric administration of YQHXR (5 ml/kg/d and 10 ml/kg/d, respectively). Animals in Sham and IVDD groups were treated with same volume of saline. All animals from different groups were sacrificed at 4-weeks after surgery, intervertebral disc tissues in each group were collected for histological analysis.

Animal experiment 2): Animals were randomly assigned to five groups: (*n* = 10 per group): Sham group, IVDD group, IVDD + YQHXR-H group, IVDD+3-MA group, IVDD + YQHXR-H+3-MA group. After induction of the IVDD model described above, rats in IVDD + YQHXR-H group were treated with intragastric administration of YQHXR-H (10 ml/kg/d). In parallel, rats in the other two groups were treated with 3-Methyladenine (3-MA) (10 mg/kg once 2 days for 2 weeks, No. HY-19312, MedChemExpress, ShangHai, China) in the absence or presence of YQHXR-H.

Animal experiment 3): Animals were randomly assigned to five groups: (*n* = 10 per group): Sham group, IVDD group, IVDD + YQHXR-H group, IVDD + Spautin-1 group, IVDD + YQHXR-H + Spautin-1 group. After induction of the IVDD model described above, rats in IVDD + YQHXR-H group were treated with intragastric administration of YQHXR-H (10 ml/kg). In parallel, rats in the other two groups were treated with Spautin-1 (7 mg/kg once 2 days for 2 weeks, No. HY-12990, MedChemExpress, ShangHai, China) in the absence or presence of YQHXR-H.

### Magnetic Resonance Imaging (MRI)

Animals were anaesthetized with sodium pentobarbital (i.p.) at a dosage of 50 mg/kg at and 4 weeks after surgery, and their tails were evaluated by MRI examination. The sagittal image of rat coccyx was recorded under T2-weighted settings using a 3.0 T clinical magnet (Philips Intera Achieva 3.0 MR) as previously reported (Shao et al., 2020). The MRIs of the position of the intervertebral disc (C4-C5) were assessed for the degree of IVDD by another blinded researcher using the Pfirrmann grading system (Pfirrmann et al., 2001).

### Histopathologic Analysis

Animals were sacrificed with 150 mg/kg sodium pentobarbital (i.p.), their tails were harvested, fixed in 4% paraformaldehyde overnight, and then decalcified with 10% EDTA. Next, all tissues used in this experiment were dehydrated, embedded in paraffin, and cut into sections with a thickness of 5 μm. The morphology of the NP and AF was investigated by hematoxylin and eosin (H&E) and safranin O Fast Green staining (Shao et al., 2020), Images were assessed by a histological researcher who was blinded (Han et al., 2008).

### Detection of Inflammatory Cytokines

Inflammatory cytokines, e.g. tumor necrosis factor-α (TNF-α, No. SEKR-0009), interleukin-6 (IL-6, No. SEKR-0002), and interleukin-1 beta (IL-1β, No. SERK-0005), were performed as the manufacturer’s instructions from Solarbio Life Science Co., Ltd. (Beijing, China), respectively.

### Transmission Electron Microscopy

Collected tissues were fixed with 2.5% glutaraldehyde (pH = 7.2) at 4°C. On the following day, after rising with PBS, samples were fixed with 1% osmium tetroxide and then stained with 2% uranyl acetate. Gradient dehydration was performed with different concentrations of ethanol and acetone. Fixed tissues were embedded into araldite and subsequently cut into semi-thin slices. The slices were stained with 3% uranium acetate—lead citrate and finally scanned by transmission electron microscopy (Hitachi, Tokyo, Japan).

### Real-Time PCR

Total RNA was extracted using the Rapid RNA extraction kit for bone tissue (No. AC1301, SparkJade Biotechnology Co., Ltd., Shandong, China) based on the manufacturer’s protocol. Then, total RNA was reverse transcribed to synthesize cDNA and amplified using the CFX96 Real-Time PCR System (Bio-Rad Laboratories, CA) and universal SYBR Green qPCR Supermix. The forward and reverse primer sequences for proteoglycan, collagen II, AMPK, VSP34, and USP13, are listed in Table 2. The housekeeping gene (GAPDH) was used as internal control. The mean cycle threshold (Ct) values of the gene were determined using the 2^−ΔΔCt^ method (Pfaffl, 2001).

**TABLE 2:** The primers for the quantitative real-time PCR.

Gene	Accession	Forward primer	Reverse primer
Proteoglycan	58,968	TAC​GAC​GCC​ATC​TGC​TAC​AC	AGT​CCA​GTG​TGT​AGC​GTG​TG
collagen II	25,412	GCC​AGG​ATG​CCC​GAA​AAT​TAG	GGC​TGC​AAA​GTT​TCC​TCC​AC
VPS34	65052	TGA​TGG​GGA​AAA​CCT​AGA​GCA​GG	TGC​ACC​AGG​CGA​TCT​ACA​AA
AMPK	65248	GGC​CTC​ACC​CTG​AGA​GAG​TA	ATG​CCA​CTT​TGC​CTT​CCG​TA
USP13	310,306	AGT​GCT​CAG​CTC​AAA​GTC​CC	CCA​GTT​GCA​CGA​GGT​TGT​TG
GAPDH	24,383	AGT​GCC​AGC​CTC​GTC​TCA​TA	GAT​GGT​GAT​GGG​TTT​CCC​GT

### Western Blotting

After extraction of total protein by RIPA buffer containing phosphatase inhibitors (Biotech Well, Shanghai, China), the concentration of proteins was measured by The BCA protein assay kit (Beyotime, Shanghai, China). Equivalent amounts of protein in each sample were separated by 8–10% sodium dodecyl sulfate-polyacrylamide gel electrophoresis (SDS-PAGE) and followed by transferred onto a polyvinylidenedifluoride (PVDF) membrane (BIO-RAD, United States). Subsequently, these protein was probed with the respective primary antibodies at 4°C overnight, including USP13 (1:1,000, No. PA5-106761, Thermo Fisher Scientific, Massachusetts, United States), AMPK (1:1,000, No. 4150, Cell Signaling Technology, Beverly, MA, United States), LC3 (1:1,000, No. 4599, Cell Signaling Technology, Beverly, MA, United States), Beclin 1 (1:1,000, No. 3495, Cell Signaling Technology, Beverly, MA, United States), VPS34 (1:1,000, No. ab124905, Abcam, Cambridge, United Kingdom) and GAPDH (1:3,000, No. 5174, Cell Signaling Technology, Beverly, MA, United States) followed by addition of appropriate HRP-conjugated goat anti-rabbit IgG (1:5,000, No. S0001, Affinity Biosciences, Beijing, China) at 37°C for 1 h. GAPDH was used as an endogenous reference. The protein signal was analyzed by ECL assay kit (enhanced). Each trial was performed in triplicate.

### Immunoprecipitation

Intervertebral disc tissue samples were treated with ice-cold IP lysis buffer on ice for 30 min, centrifuged at 12,000 g for 30 min. The supernatant of the lysate was collected. About 1 μg protein antibody and 50 μL protein A-agarose beads were added to the appropriate amount of lysate and incubated at 4°C overnight. Afterwards, the agarose beads were centrifuged to the bottom of the tube at 4°C, 3,000 rpm for 3 min. The supernatant was extracted out and the agarose beads were washed 3–4 times with 1 ml lysis buffer. Lysis buffers were carefully collected and added 2 × SDS loading buffer and boiled for 10 min. Finally, western blot was used for analysis.

Mouse Anti-rabbit IgG (Conformation Specific) (1:5,000, No. 5127, Cell Signaling Technology, Beverly, MA, United States) was used as second antibody.

### Statistical Analysis

Data were shown as mean ± standard deviation (SD). One-way ANOVA and Donnet’s post-analysis or Tukey’s multiple comparison test were used to evaluate the differences among groups (Software: Graphpad prism 8.0). The value less than 0.05 indicated statistical significance.

## Results

### Yiqi Huoxue Recipe was Helpful to Weight Gain and Suppress Inflammation in Needle-Punctured Rats

**FIGURE 2:**
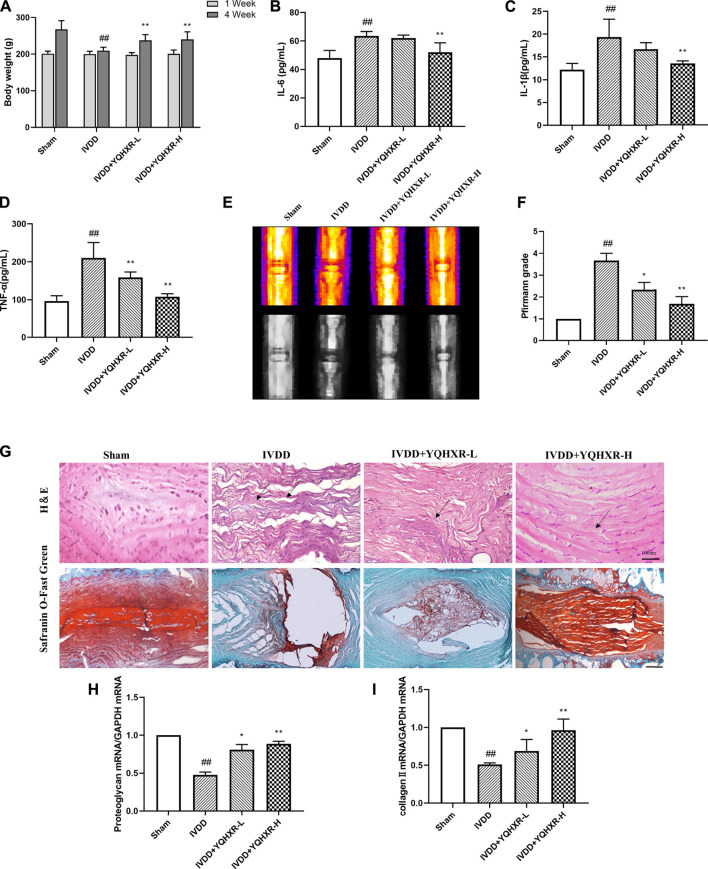
YQBXR ameliorated IVDD progression in needle-punctured rat model. **(A)** The body weight of IVDD rats. **(B–D)** Levels of inflammatory factors in serum. **(E)** Representative images of IVDD rat tails on T2-weighted MRI at 4-weeks post-surgery. **(F)** Pfirrmann scores of MRI. **(G)** Representative images of the degenerative disc at 4-weeks post-surgery by H&E and Safranin O Fast Green staining. **(H–I)** Real-time assay was performed to evaluate the fibrosis of the degenerative disc in different groups. The data represent the mean ± SD. Significant differences are shown as ^##^ *p* < 0.01 vs Sham group; *p < 0.05, **p < 0.01 vs IVDD group.

To explore whether YQHXR exerted a protective effect on IVDD *in vivo*, we established a tail needle-punctured IVDD model with administration of YQHXR. Firstly, we measured the effect of YQHXR on the weight of rats (Figure 2A). Compared with Sham group, the weight of rats in IVDD group increased slowly at the fourth week, and the statistical analysis showed a significant difference (*p* < 0.01), while YQHXR-treated groups showed obvious weight gain compared with IVDD group (*p* < 0.01). The levels of inflammatory factors (IL-1β, TNF-α, and IL-6) of IVDD group were much higher than those of Sham group, which were suppressed by YQHXR administration (*p* < 0.01, Figures 2B–D).

### Yiqi Huoxue Recipe Delayed the Progression of Intervertebral Disc Degeneration in Needle-Punctured Rats

To evaluate the degree of intervertebral disc degeneration in rats of different groups, MRI examination and Pfirrmann score (Figures 2E,F) were performed at 0 and 4 weeks after puncture, in which higher scores indicated increased severity of IVDD. Compared with Sham group, MRI-calculated signal intensity of IVDD rats significantly increased (*p* < 0.05), whereas IVDD rats treated with YQHXR displayed much lower signal intensity (*p* < 0.05 or *p* < 0.01).

Moreover, the protective effect of UA on intervertebral disc after 4 weeks of surgery was further confirmed by H&E and Safranin O Fast Green staining (Figure 2G). The results indicated that the NP cells in the intervertebral disc of Sham group were evenly dispersed in the extracellular matrix. The arrangement of AF tissue around the NP was normal, without obvious rupture or disorder. Compared with Sham group, the NP cells in IVDD group presented obvious shrinkage, and the arrangement of AF was disordered. In contrast, the uniformity of AF in the treatment groups of YQHXR was more orderly, the fracture was much milder than that in IVDD group, and some nucleus pulposus cells were lost. Especially, as shown in Figure 2G (lower panel), in IVDD group, the red positive tissue representing the proteoglycan matrix was obviously less compared with Sham group. However, YQHXR partially protected tissues against fibrosis, which were also proved by Real-time PCR analysis (Figures 2H,I). These results indicated that YQHXR treatment markedly reduced the disruption of the disc structure and fibrosis.

### Yiqi Huoxue Recipe Activated Autophagy of the Degenerated Disc to Attenuate the Progression of Intervertebral Disc Degeneration

**FIGURE 3:**
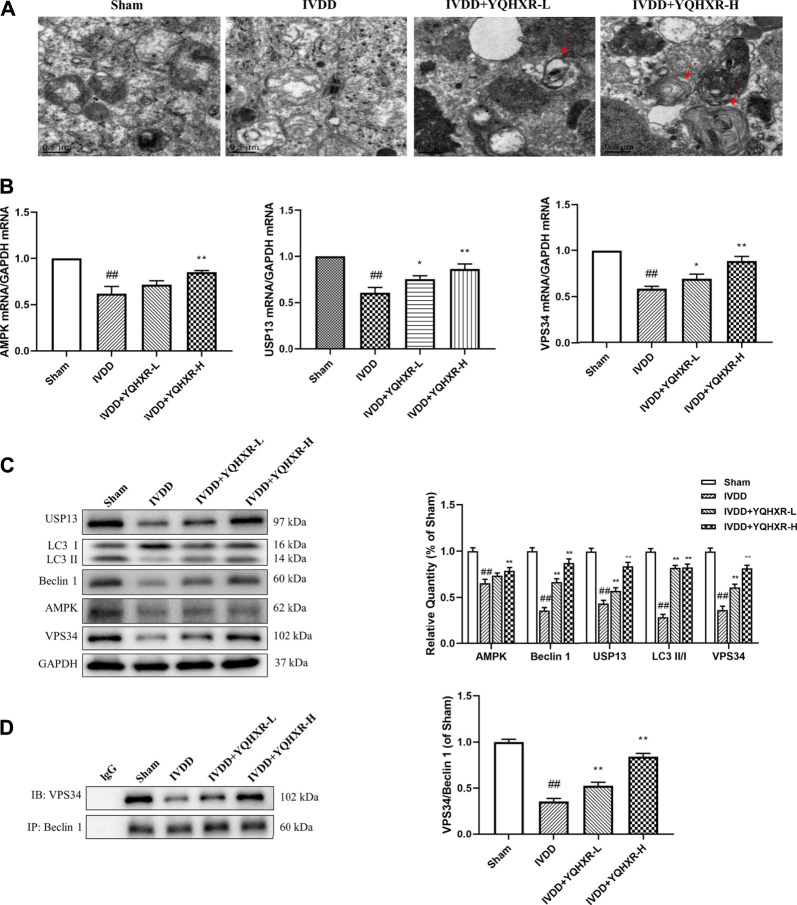
Autophagy was activated by YQBXR administration in needle-punctured IVDD rats. **(A)** Transmission electron microscope analysis of rat discs (red arrows: autophagosomes or autolysosomes, scale bar: 0.5 μm). **(B)** The mRNA levels of autophagy pathway-related gene by real-time PCR analysis. **(C–D)** Representative bands of autophagy pathway-related proteins by western blotting and immunoprecipitation analysis, and their quantitative results. The data represent the mean ± SD. Significant differences are shown as ^##^ *p* < 0.01 vs Sham group; *p < 0.05, **p < 0.01 vs IVDD group.

It has been reported that autophagy is attenuated in human degenerated disc tissues. (Chen et al., 2020). Therefore, we clarified whether YQHXR contributed to autophagy involved in the degeneration of discs. Firstly, by using transmission electron microscopy, we found that autophagy was attenuated in the IVDD rat model. However, the numbers of autophagosomes and autolysosomes were significantly increased by YQHXR treatment compared with sham surgery or punctured rats (Figure 3A).

Next, we sought to explore how YQHXR induced autophagy to alleviate IVDD using real-time PCR, western blot, and immunoprecipitation analysis (Figures 3B–D). YQHXR might attenuate the progression of IVDD through activation of AMPK/VPS34 (Class III phosphatidylinositol 3-kinase, PI3KC3/VPS34) pathway. As expected, the gene and expression levels of AMPK and VPS34 in IVDD group were much lower than those in Sham group, which can be increased by YQHXR (*p* < 0.01). However, YQHXR had no effect on Beclin1 gene level (not shown), whereas significantly increased its protein expression. The possible reason was that the transcription and translation of USP13 were regulated by YQHXR. In addition, the expression levels of USP13, Beclin1, and LC3 increased in a dose dependent manner of YQHXR compared with IVDD group (*p* < 0.01). The major components of core complex of PI3KC3 consist of VPS34-Beclin 1 (Boukhalfa et al., 2020). Results from both western blot and immunoprecipitation indicated that YQHXR induced autophagy through promotion of the formation of VPS34-Beclin one complex, further enhancing LC3 expression.

### Yiqi Huoxue Recipe Promoted the Formation of Beclin1-VPS34 Complex to Activate Autophagy

**FIGURE 4:**
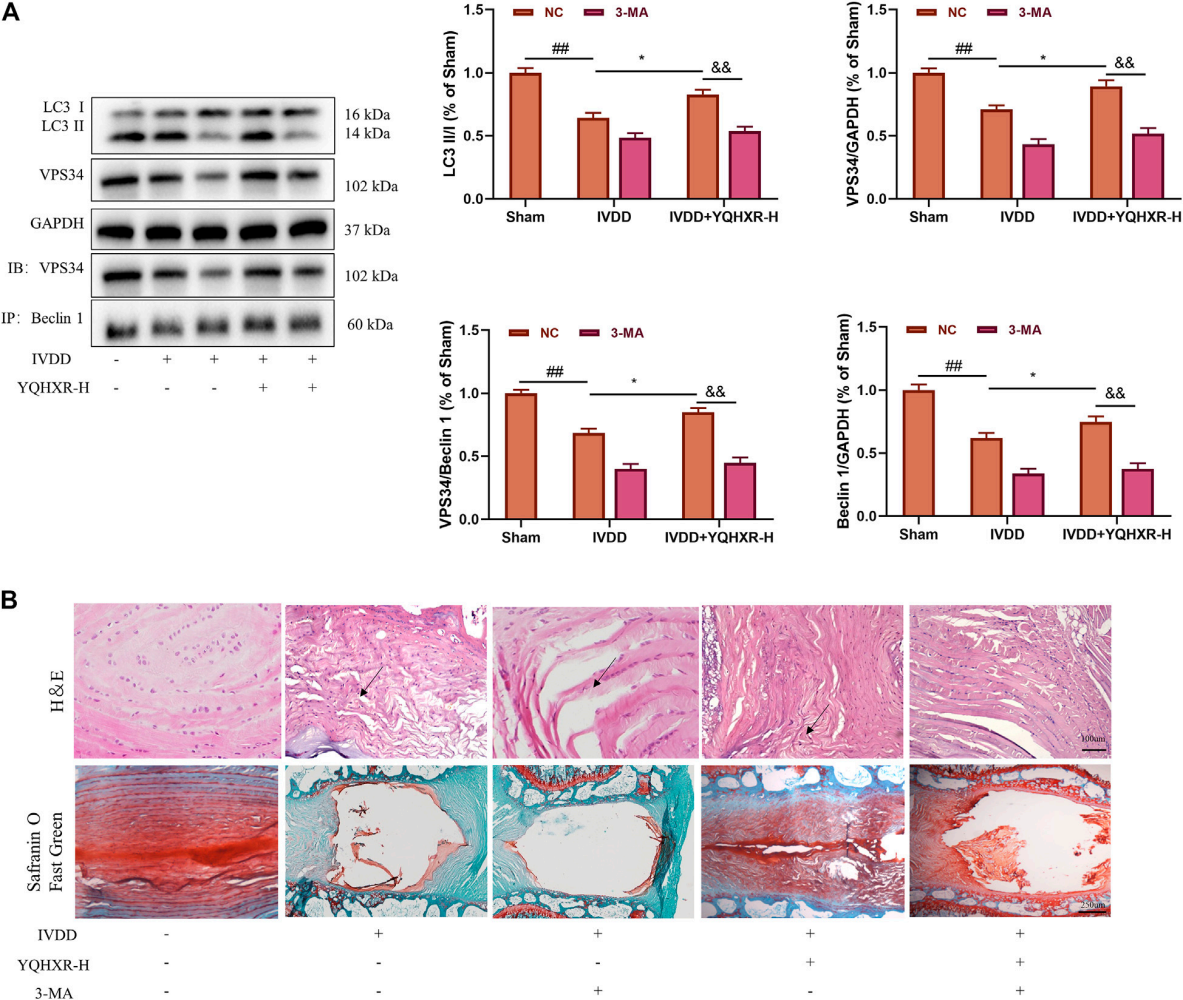
YQHXR promoted VPS34 expression in the formation of Beclin1-VPS34 complex. **(A)** The effect of YQHXR on the formation of Beclin1-VPS34 complex in the absence or presence of 3-MA was analyzed by western blotting and immunoprecipitation analysis. **(B)** The effect of YQHXR on the pathological changes of IVDD rats in the absence or presence of 3-MA. The data represent the mean ± SD. Significant differences among different groups are shown as ^##^ *p* < 0.01 vs Sham + NC group; *p < 0.05 vs IVDD + NC group; ^&&^ *p* < 0.01 vs IVDD + YQHXR-H + NC group.

**FIGURE 5:**
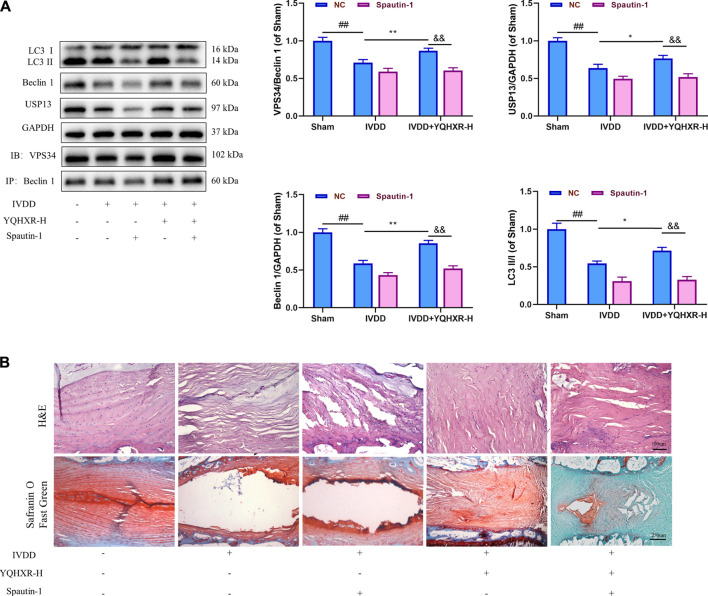
YQHXR increased the protein level of the ubiquitinase USP13 for the inhibition of Beclin1 degradation. **(A)** The effect of YQHXR on stabilization of Beclin1-VPS34 complex in the absence or presence of Spautin-1 was analyzed by western blotting and immunoprecipitation analysis. **(B)** The effect of YQHXR on the pathological changes of IVDD rats in the absence or presence of Spautin-1. The data represent the mean ± SD. Significant differences among different groups are shown as ^##^ *p* < 0.01 vs Sham + NC group; *p < 0.05, **p < 0.01 vs IVDD + NC group; ^&&^ *p* < 0.01 vs IVDD + YQHXR-H + NC group.

We have previously mentioned the hypothesis that YQHXR induced autophagy through promotion of the formation of VPS34-Beclin one complex. To test this hypothesis further, 3-MA (an PI3K inhibitor against VPS34) or Spautin-1 (deubiquitination inhibitor against USP13) was used to inhibit the autophagy-related pathway in IVDD rats. We observed that both 3-MA and Spautin-1 compromised the increased expression of Beclin1-VPS34 complex by YQHXR in IVDD rats, respectively (*p* > 0.05, Figure 4A and Figure 5A). Due to 3-MA or Spautin-1 intervention, YQHXR exerted no beneficial therapeutic effect on the loss of NP cells and rupture of AF tissue of the intervertebral disc (Figure 4B and Figure 5B).

All in all, YQHXR promoted the formation of Beclin1-VPS34 complex to activate autophagy through not only activation of the upstream protein AMPK but also upregulation of the ubiquitinase USP13.

## Discussion

IVDD is the main cause of spinal degenerative diseases, the pathological change of IVDD includes disc herniation, low back pain, spinal canal stenosis, scoliosis, and osteophyte formation (Adams and Roughley, 2006; Anirudh, 2018). IVDD is a widespread pathogenic process, during which NP cells degenerate and disappear in varying degrees, and the AF tissues of the intervertebral disc appear abnormal and rupture, afterwards, NP tissues protruded from the posterior or spinal canal from the rupture, resulting in stimulation or compression of adjacent spinal nerve roots. It not only brings great pain to patients, but also causes large social and economic burden (Wai et al., 2010). Therefore, novel drugs or treatment strategies which can effectively alleviate IVDD are urgently needed.

The composition of YQHXR skillfully combines the therapeutic principles of nursing vitality (tonifying Qi), promoting blood circulation, removing blood stasis, and dredging collaterals. In the recipe, *Astragalus* mongholicus Bunge is used to replenish the Qi of spleen and stomach, so that Qi and blood can be unblocked and blood stasis can be removed without damaging the body; Angelica sinensis (Oliv.) Diels is also used to promote blood circulation, remove blood stasis and dredge collaterals. Early clinical investigation confirmed that YQHXR promoted the reabsorption of ruptured lumbar disc herniation (Yu et al., 2015). However, the precise molecular mechanisms of YQHXR against IVDD pathogenesis remain unclear.

Previous studies have shown that intervertebral disc degeneration is a complex process of multi-factor interaction, in which a variety of cytokines are involved in the pathological progression of IVDD, e.g. IL-1β, TNF-α, IL-6 (Wang et al., 2020). Estrogen effectively alleviated IVDD development by suppressing cell death of disc in multiple ways, including the inhibition of the inflammatory cytokines (IL-1β, TNF-α) and promoting autophagy. In our study, YQHXR also suppressed the inflammatory reaction by decreasing the release of inflammatory cytokines (IL-1β, TNF-α, and IL-6).

In recent years, increasing evidence have indicated that autophagy plays a key role in IVDD (Chen et al., 2014). Class III phosphatidylinositol 3-kinase (PI3KC3) have been confirmed pleiotropically involved in autophagy pathways. The major components of human core complex of PI3KC3 consist of PI3KC3/VPS34, p150 and Beclin 1 (Boukhalfa et al., 2020). VPS34 catalyzes the phosphorylation of phosphatidylinositol (PI) to produce 3-phosphate phosphatidylinositol (PI3P), which is necessary for the formation of autophagosome (Boukhalfa et al., 2020). So far, studies have shown that the formation of VPS34 complex differentially regulated by AMPK is the main mechanism of activating autophagy (Kim et al., 2013; Kim and Guan, 2013). In addition, the increased activity and expression level of deubiquitinases can stabilize Beclin1-VPS34-p150 complexs, such as USP13 (Liu et al., 2011; Kim, 2012). Western blotting results in animal experiments showed that in IVDD group, the expressions of AMPK and USP13 were inhibited compared with Sham group, followed by suppressing the formation of Beclin1-VPS34-p150 complex and its related protein levels, which was reversed by different dosages of YQHXR. Furthermore, we found YQHXR promoted the appearance of autophagosomes under transmission electron microscopy.

In order to further explore how YQHXR affected the autophagy pathway to alleviate IVDD, 3-MA (PI3K inhibitor against VPS34) or Spautin-1 (deubiquitination inhibitor against USP13) was used to inhibit the autophagy-related pathway in IVDD rats. We observed that both 3-MA and Spautin-1 cancelled the increased expression of Beclin1-VPS34 complex by YQHXR in IVDD rats, which further indicated YQHXR promoted the formation of Beclin1-VPS34 complex to activate autophagy through activation of the upstream protein AMPK and upregulation of ubiquitinase USP13 expression, thus in turn alleviating the development of IVDD.

In our study, YQHXR could indeed delay the pathological process of IVDD in rats, including inhibiting the release of inflammatory factors. However, the clinical application of YQHXR in the treatment of IVDD is still in its infancy. Further studies on the molecular biological mechanism of YQHXR will be helpful to explore its potential medical value in delaying or reversing the progress of IVDD.

There are some limitations of the study. First, the IVDD model was established only in C4-5 tail intervertebral disc. Comparison between different levels with different treatment performed should be evaluated. Second, although we have found that AMPK is involved in the anti-IVDD effect induced by YQHXR, the intervention of AMPK is needed to elucidate its role in YQHXR-induced protective effect in IVDD. Third, given YQHXR-L only rescued partial symptoms caused by IVDD, which might be related to inadequate time interval for the treatment of IVDD. Hence, effect of longer YQHXR administration on IVDD should be assessed.

**FIGURE 6:**
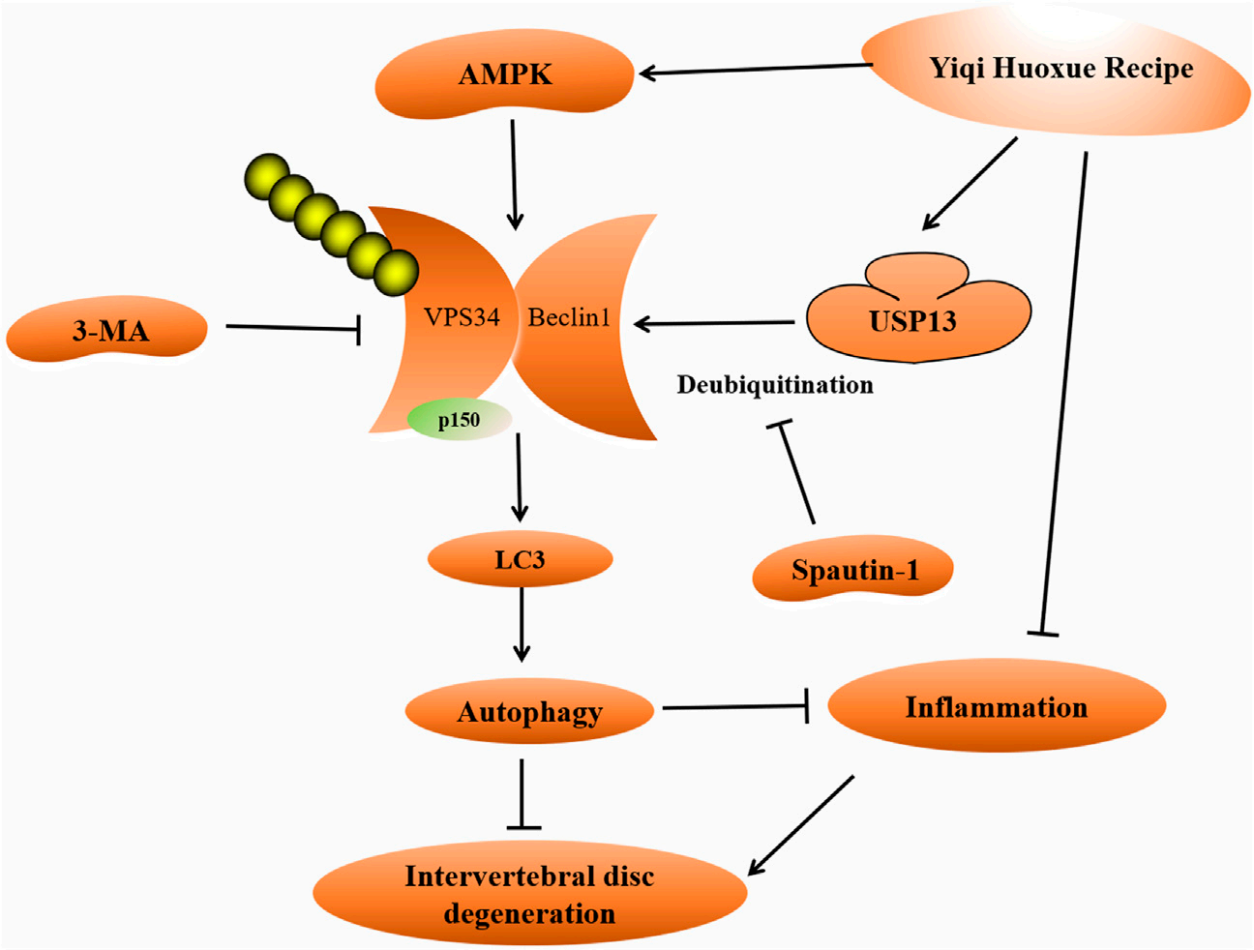
Schematic model of the proposed signaling pathways of the effect of YQHXR in IVDD.

In conclusion, we found that YQHXR-induced autophagy attenuated the release of inflammatory factors. In addition, YQHXR promoted the formation of Beclin1-VPS34 complex to activate autophagy through activation of the upstream protein AMPK and upregulation of the ubiquitinase USP13. We proposed the potential molecular mechanism of YQHXR on autophagy for the first time, so as to provide a theoretical and experimental basis for the clinical application of YQHXR in the treatment of IVDD-related diseases (Figure 6).

## Data Availability

The original contributions presented in the study are included in the article/Supplementary Material, further inquiries can be directed to the corresponding author.
